# Correction: Glycal mediated synthesis of piperidine alkaloids: fagomine, 4-*epi*-fagomine, 2-deoxynojirimycin, and an advanced intermediate, iminoglycal

**DOI:** 10.1039/d2ra90122f

**Published:** 2022-12-01

**Authors:** Hemender R. Chand, Mrityunjay K. Tiwari, Asish K. Bhattacharya

**Affiliations:** Division of Organic Chemistry, CSIR-National Chemical Laboratory Dr Homi Bhabha Road, Pashan Pune Maharashtra 411008 India ak.bhattacharya@ncl.res.in; Academy of Scientific and Innovative Research (AcSIR) Ghaziabad India; Physical and Materials Chemistry Division, CSIR-National Chemical Laboratory Dr Homi Bhabha Road, Pashan Pune Maharashtra 411008 India

## Abstract

Correction for ‘Glycal mediated synthesis of piperidine alkaloids: fagomine, 4-*epi*-fagomine, 2-deoxynojirimycin, and an advanced intermediate, iminoglycal’ by Hemender R. Chand *et al.*, *RSC Adv.*, 2022, **12**, 33021–33031, https://doi.org/10.1039/D2RA05224E.

The authors regret that one of the authors’ names, Mrityunjay K. Tiwari, was spelt incorrectly in the original manuscript. The corrected list of authors for this paper is as shown above. The authors regret that affiliation c was incorrectly shown in the original manuscript. The corrected list of affiliations is as shown below.

The Royal Society of Chemistry apologises for these errors and any consequent inconvenience to authors and readers.

## Supplementary Material

